# How is post-industrial decline associated with the geography of physical activity? Evidence from the Health Survey for England

**DOI:** 10.1016/j.socscimed.2013.12.004

**Published:** 2014-03

**Authors:** Esther Rind, Andy Jones, Humphrey Southall

**Affiliations:** a School of GeoSciences, University of Edinburgh, Drummond Street, Edinburgh EH8 9XP, UK; b Norwich Medical School, University of East Anglia, UK; c Department of Geography, University of Portsmouth, UK

**Keywords:** England, Physical activity, Geography, Deindustrialisation, Multilevel analysis

## Abstract

In recent decades, the prevalence of physical activity has declined considerably in many developed countries, which has been related to rising levels of obesity and several weight-related medical conditions, such as coronary heart disease. There is evidence that areas exhibiting particularly low levels of physical activity have undergone a strong transition away from employment in physically demanding occupations. It is proposed that such processes of deindustrialisation may be causally linked to unexplained geographical disparities in physical activity. This study investigates how geographical variations in deindustrialisation are associated with current levels of physical activity across different activity domains and relevant macro-economic time periods in England. The analysis includes data on 27,414 adults from the Health Survey for England 2006 and 2008 who reported total, occupational, domestic, recreational and walking activity. Based on employment change in industries associated with heavy manual work, a local measurement of industrial decline was developed, covering the period 1841–2001. We applied a multilevel modelling approach to study associations between industrial decline and physical activity. Results indicate that the process of deindustrialisation appears to be associated with patterns of physical activity and that this is independent of household income. The effects observed were generally similar for men and women. However, the nature of the association differed across areas, time periods and employment types; in particular, residents of districts characterised by a history of manufacturing and mining employment had increased odds of reporting low activity levels. We conclude that post-industrial change may be a factor in explaining present-day variations in physical activity, emphasising the plausible impact of inherited cultures and regional identities on health related behaviours.

## Highlights

• This study is the first to link different physical activity domains to the historical context of deindustrialisation
• We found some evidence that processes of deindustrialisation may be associated with lower physical activity
• Lower overall activity was generally seen in areas with historically high employment then decline in manufacturing and mining
• Findings suggest cultures of non-participation in physical activity may persist in areas experiencing losses in manual jobs

## Introduction

Achieving a health-promoting weekly energy expenditure from physical activity (PA) of about 1000 kcal equates to just 1 h of moderate walking five days a week (Warburton, Nicol, & Bredin, 2006a, 2006b). However, in Europe more than 65% of the adult population is insufficiently active, and this has been related to increasing levels of obesity and related diseases such as diabetes, heart disease and several forms of cancer (World Health Organisation, 2006). Compared to many other European countries, the proportion of sedentary residents in the UK is relatively high and the proportion of the population being sufficiently active is low (Sjöström, Oja, Hagströmer, Smith, & Bauman, 2006). The average person walks less than a mile per day, and between 1975 and 2009 the average annual walking distance decreased by 68 miles, a decline of 26% (Department for Transport, 2010; Fox & Hillsdon, 2007).

Recently, Stamatakis, Ekelund, and Wareham (2007) investigated temporal trends in PA in England and showed that levels of occupational PA have decreased significantly between 1991 and 2004. There was a small increase in recreational PA across the country during this period, but this was not pronounced in men from manual social classes, lower income households, and those from non-white ethnic backgrounds (Stamatakis & Chaudhury, 2008). This is of particular concern given that these groups are the ones who are most vulnerable to the loss of PA from their occupation (Popham & Mitchell, 2007).

Using data from the Health Survey for England (HSE) 2003, Allender, Foster, and Boxer (2008) have shown that occupational activity significantly contributes to overall PA in English adults. Further, the contribution of occupational activity is shown to be socially patterned, with both men and women in manual occupations being more likely to meet the government recommended PA guidelines than their non-manual counterparts. Those in manual occupations, however, show lower levels of recreational activity (Kirk & Rhodes, 2011).

It has been argued that the lower prevalence of recreational activity in manual classes could persist even after a transition to less physically demanding occupations (Jones & Bentham, 2009), resulting in low levels of overall PA in individuals and communities who have been particularly affected by industrial transitions. Observed decreases in occupational PA in England, along with many other European countries, have been related to industrial decline and technical progress including processes of automatisation and computerisation. A consequence has been a considerably reduced need for heavy manual work, even in areas where employment in heavy industries has remained relatively high (Bazen & Thirlwall, 1997). In England, the demand for manual employment has long been in decline and over the last century this has been particularly so in the agricultural sector (−92%), manufacturing (−51%) and the mining industry (−90%) (Southall & Great Britain Historical GIS Project, 2009).

There is overwhelming evidence that health outcomes and behaviours, including PA, mirror levels of socio-economic deprivation and inequality in ways that cannot be solely explained by the individual characteristics of the population under study (e.g. Cleland et al., 2010; Doran, Drever, & Whitehead, 2004; Krieger, 2001). Doran, Drever, and Whitehead (2006) for example showed that mining, manufacturing and other industrial areas had lower life expectancies than predicted by their level of deprivation. Related to such observations, there is some evidence that processes associated with industrial decline may directly impact these health and related behaviours. For example, Mitchell, Gleave, Bartley, Wiggins, and Joshi (2000) investigated the effect of deindustrialisation on self-reported physical health in Great Britain between 1981 and 1991. They found that residents living in areas that were highly dependent on industrial employment and that had experienced high levels of employment decline were more likely to report physical ill-health compared to those where the effects of industrial decline were less severe.

In terms of PA, prior research has shown that residents of more northerly and urban districts that have undergone a particular strong transition from industrial to postindustrial economies are more likely to report low levels of PA than their more southerly and rural counterparts (Blaxter, 1990; Ellis, Grimsley, Goyder, Blank, & Peters, 2007; Rind & Jones, 2011). Indeed we have previously described substantial geographical variations in recreational PA in England (Rind & Jones, 2011). More recently we have developed a conceptual framework that describes how unexplained components of these variations might be associated with industrial decline in parts of the country (Rind & Jones, in press). In that framework, we illustrate how decreases in occupational PA might be coupled with other local changes including the loss of social networks, rising levels of social fragmentation, and degradation of the physical environment to create obesogenic environments in areas that have experienced losses in employment in manual occupations. We note the need to empirically search for empirical evidence regarding the potential mechanisms outlined in the framework.

Industrial decline catalysed the transformation of occupational structures, the widening of health inequalities and has been one of the most incisive contextual large-scale drivers of social change in modern history. However, there has been rather little research attempting to assess how patterns of deindustrialisation may affect observed variations in the prevalence of PA across the different activity domains. This study has been undertaken to investigate associations between individual and contextual correlates of PA related to the context of industrial decline, and in doing so to test for evidence of the processes outlined in the conceptual framework developed in Rind and Jones (in press). We hypothesise that levels of PA are likely to be low in areas where there has been a history of high employment as well as high decline in occupations associated with heavy manual work. We further hypothesise the relationship between deindustrialisation and PA to vary according to macro-economic change. We hope that the outcomes of this study will contribute to a better understanding of where and how factors related to changes in socio-economic conditions affect levels of PA across the different activity domains.

## Methods

### Data

The primary data sources for this study were the Health Surveys for England (HSE) 2006 and 2008 (NHS Information Centre, 2011), as well as the Great Britain Historical GIS project (Gregory & Southall, 1998; Southall, 2011). Subsequently, the use of these sources to provide the outcomes and exposure measures analysed is described.

### Physical activity

The HSE is an annual survey drawn from a nationally representative general population sample that includes data on several indicators of health and related behaviours. The full sampling methodology and the derivation of the PA variables is described elsewhere (Craig & Mindell, 2008; Craig, Mindell, & Hirani, 2009). Briefly, a random sample of core addresses are selected from the Postcode Address File and households are sampled proportionately across the nine Government Office regions of England. For the survey conducted in 2006, 14,142 adults aged 16 and over were interviewed at their homes, and 15,102 were interviewed for the HSE 2008. All PA measures recorded and used for this study were based on self-report. The HSE samples for 2006 and 2008 include comparable measurements of total, occupational, domestic, recreational, and walking activities, and these two years were combined to provide the outcome dataset for this analysis. For this study, all participants aged 16 and above providing information for the five activity domains were identified, and individual records were obtained.

The outcome variables provided in the HSE dataset covered the frequency and intensity of activity undertaken in five different domains of PA (total PA, walking, occupational, domestic, and recreational PA) based on reported activity spells of at least 30 min duration undertaken over the last four weeks prior to interview. Total PA in both HSE surveys was measured as the number of days per week of any moderate and vigorous activity. Overall activity levels for each respondent were categorised as ‘less active’ (<1 day/week), ‘moderately active’ (1–4 days/week), or ‘highly active’ (5 or more days/week). Levels of walking activity were classified accordingly based on the reported number of days over the last 4 weeks respondents walked at a fast or brisk pace.

The intensity of reported activities for the other three domains was classified based on MET (metabolic equivalent) intensities categorised by the Compendium of Physical Activities (Ainsworth et al., 2000). One MET is considered a resting metabolic rate obtained during quiet sitting. If respondents did not report any activity of at least 30 min duration in a domain they were categorised as ‘inactive’. The classification of occupational activity levels included consideration of the respondents' reported working status, perceived intensity levels and the type of occupation according to the Standard Occupational Classification (Office for National Statistics, 1990). For domestic activities, participants were given examples of light, moderate and heavy housework, gardening, and home–improvement activities, and were asked to select those that equated most closely to their own participation. The intensity levels of recreational activities considered the type and perceived effort level. A low or high perceived effort level was defined according to not being or being out of breath during exercise. Based on estimated MET values, each respondent is classified as ‘light’ (<3 METs), ‘moderate’ (3-6 METs) and ‘vigorous’ (>6 METs) for each domain (Craig et al., 2009) for these three domains in the HSE dataset. For clarity, we renamed these original HSE headings ‘non-active, ‘less active’, ‘moderately active’ and ‘highly active’ in this article. In the two surveys utilised, there were only 70 men and 2 women who coded as reporting ‘high’ occupational PA. Hence, these cases were combined with those reporting moderate activities.

Each HSE record included a year 2001 Local Authority District (LAD) of residence identifier. LADs are administrative areas of local government in England, with an average population of 139,000 residents. In 2001, there were 354 English Local Authorities, including non-metropolitan districts, metropolitan districts, unitary authorities and London boroughs (Office for National Statistics, 2001). The HSE excluded any respondents from the City of London (in 2008) and the Isles of Scilly and Berwick-upon Tweed (both 2006 and 2008) due to the small residential populations of these LADs. In England, LADs provide a suitable scale to study variations in health outcomes, as they are large enough to provide adequate study power and can be used to represent labour market attributes such as employment change (Chevan & Stokes, 2000; Mitchell et al., 2000). The LAD identifier allowed the record to be supplemented with area-based data on industrial decline from the Great Britain Historical GIS project.

### Industrial decline

The Great Britain Historical GIS project was instigated to integrate data from a range of surveys in Britain including travel writing, census results and historical maps (Southall, 2007, 2011). The project aimed not simply to assemble a diverse range of statistical data from the full range of censuses 1841–2001, but also to enable comparisons of areas to be made over long periods by re-organising data to standardised reporting areas and topics (Gilbert & Southall, 2000). Given the wide range of classifications used by the census, standardisation by topic generally means assembling historical categories into simplified aggregate categories (Southall, 2011), and even then caveats are needed, such as miners not being separately identifiable in the 1981 Small Area Statistics.

Standardisation by area involved three distinct strategies. Firstly, 2001 “Key Statistics” were used unchanged, covering Great Britain using 408 districts and unitary authorities. Secondly, for 1971, 1981 and 1991 ward-level Small Area Statistics were assembled into the same 408 LADs using the Linking Censuses through Time system, which assigns all data for a ward to the 2001 LAD containing its geometric centre (Pateman, 2011). Thirdly, for all earlier census dates, geography conversion tables (Simpson, 2002) were constructed using parish-level census population data and detailed parish boundary mapping constructed by the project Geographical Information System (GIS) to estimate what proportion of each historical district's population should be assigned to each 2001 LAD. The geographical standardisation of the historical census data permitted the analysis of spatial change in economic outcomes across different time periods in relation to individual PA outcomes.

For this study we used data comprising, for each LAD in England, long term trends in employment data for several economic sectors, including manufacturing, mining, and agriculture for the period 1841 to 2001 (Southall & Great Britain Historical GIS Project, 2009). Following a similar methodology to that applied by Mitchell et al. (2000) in their analysis of industrial change and self-rated health, we developed a suite of variables representing industrial decline. Prior research has shown that occupation characteristics have relatively strong associations with PA (Kirk & Rhodes, 2011). Therefore, our measures were based on employment change in employment types associated with heavy manual work. Employment in manufacturing, mining and agriculture was selected as PA from these activities is likely to have moderate to vigorous intensities; the average intensity of agricultural activities is 4.3 METs, that of coal mining is 6.5 METs, and work intensities related to the manufacturing sector, such as working in a steel mill, have 7.3 METs (Ainsworth et al., 2000).

To investigate whether different macro-economic periods have a different relationship with current levels of PA we chose three different time periods to measure changes in employment. The first period, 1841 to 2001, encompassed employment change in each sector over the whole timescale for which data was available. For employment in manufacturing and mining the years from 1841 to 1971 capture employment change prior to severe industrial decline, including the closure of most of England's coal mines in the 1980s, whilst for agricultural employment that transition occurred earlier (Castells & Aoyama, 1994). Hence, the second time period covered 1841–1971 for manufacturing and mining, with 1971–2001 comprising the third. For agriculture the second period was 1841–1931, and the third 1931–2001.

Across all three employment sectors, the measurements of industrial decline used in analysis combined the level of initial employment, defined as the percentage of the LAD population employed in each of the three employment types at a particular year, with the level of subsequent employment change. As may be expected, for most LADs, employment change in manufacturing, mining and agriculture was dominated by decline. There were, however, some areas of growth. For manufacturing (1971–2001) growth was recorded for just 6 LADs, containing a total of 296 HSE respondents and for agriculture (1931–2001) there were 384 HSE respondents in 5 LADs. For all other measurements of industrial decline, the number of respondents in areas of growth ranged from 870 in 16 LADs (mining 1841–2001) to 18,285 in 251 LADs (manufacturing 1841–1971).

For the purposes of analysis, we classified each LAD using a 5-category variable for each employment type: 1) high initial employment/high decline, 2) high initial employment/low decline, 3) low initial employment/high decline, 4) low initial employment/low decline, and 5) growth. ‘High’ or ‘low’ initial employment was defined according to whether each LAD was above or below the median initial employment level for each period. Employment change was measured by calculating the percentage change during each time period, and again ‘high decline’ and ‘low decline’ were based on whether an LAD fell above or below the median. The resulting values were mapped using the ArcGIS 9.3 Geographical Information System (ESRI Inc.).

### Individual covariates

Information on a range of individual covariates that may be associated with PA (Gidlow, Johnston, Crone, Ellis, & James, 2006; Trost, Owen, Bauman, Sallis, & Brown, 2002) were available from the HSE and those included in the analyses were age in years, sex, ethnicity (White vs. non-White), presence of long term limiting illness (yes vs. no), and self-assessed general health (very good/good vs. fair vs. bad/very bad). In addition, we had information on equalised household income for 22,066 subjects for both of the survey years (2006/2008). Quintiles of this categorical measure ranged from <£10,598 to ≥£40,373.

### Analyses

Rank correlation [Spearman's rho (*ρ*)] was used to explore associations between levels of total PA and the other activity domains. Including a measure of effect direction (gamma coefficient), we calculated Pearson's Chi-square tests to test unadjusted associations between levels of PA and the magnitude of employment change in each sector. Results were stratified by sex as it was anticipated that employment decline in heavy manual work may have a stronger effect on men than on women.

To investigate the relationship between PA and industrial decline, we fitted applied multilevel ordinal regression models using a two level structure of individuals (level 1) nested within districts (level 2). Ordered proportional odds models applying a logit link function were used since this procedure allows the derivation of interpretable odds ratios (Garson, 2009). The coding of the outcome variables was ascending, for example total PA: 1 = low, 2 = medium, 3 = high, so we modelled the log odds of low PA versus medium and high PA. In addition to testing the significance of individual coefficients, we tested for trends in association across the categories of industrial decline by modelling the categorical variable as continuous.

The modelling procedure comprised three steps. For each of the five PA outcomes, the first model included the all the individual covariates and in the next stage we added our measurement of industrial decline for each employment type at each of the three time periods. In the third stage we then fitted an additional term to each model, equalised household income, to see if controlling for individual socioeconomic circumstances attenuated associations with area measures amongst the subset of the sample for which income data was available. Although energy expenditure from employment differs by sex, the fitting of the interaction terms in the regression models showed no consistent differences in association by gender, and therefore the models were not sex stratified. All regression models were fitted in MLwiN 2.23 (Rasbash, Charlton, Browne, Healy, & Cameron, 2011), whilst other analyses were undertaken in SPSS version 16 (SPSS Inc). This study was approved by the Research Ethics Committee of the University of East Anglia.

## Results

In total, the records of 29,244 respondents to the 2006 and 2008 HSE were obtained. Of these, 1830 (6.3%) were excluded due to missing data on one or more domains of PA. Hence, the final sample analysed included 27,414 adults. Of the included sample, 45% were male (compared to 49% in the mid-year 2007 population estimates for England; Office for National Statistics, 2010), 27% were aged under 35 (compared to 24%), and 18% were aged over 64 (compared to 19%). Some 90% (compared to 88%) gave their ethnic origin as White. In total, 24% reported some limiting long-term illness, and 75% reported their health as ‘very good’ or ‘good’. Just 7% reported their health as ‘poor’. Table 1 summarises the PA and individual characteristics of the included sample.

Furthermore, all of the PA domains were positively associated with each other. For men, levels of total PA showed the strongest association with levels of walking (*ρ* = 0.514), followed by occupational (*ρ* = 0.434), recreational (*ρ* = 0.380) and domestic activities (*ρ* = 0.301). For women, levels of total PA also had the strongest association with levels of walking (*ρ* = 0.585), followed by domestic (*ρ* = 0.397), recreational (*ρ* = 0.387) and occupational activities (*ρ* = 0.324) (all *p* < 0.001).

**Fig. 1:**
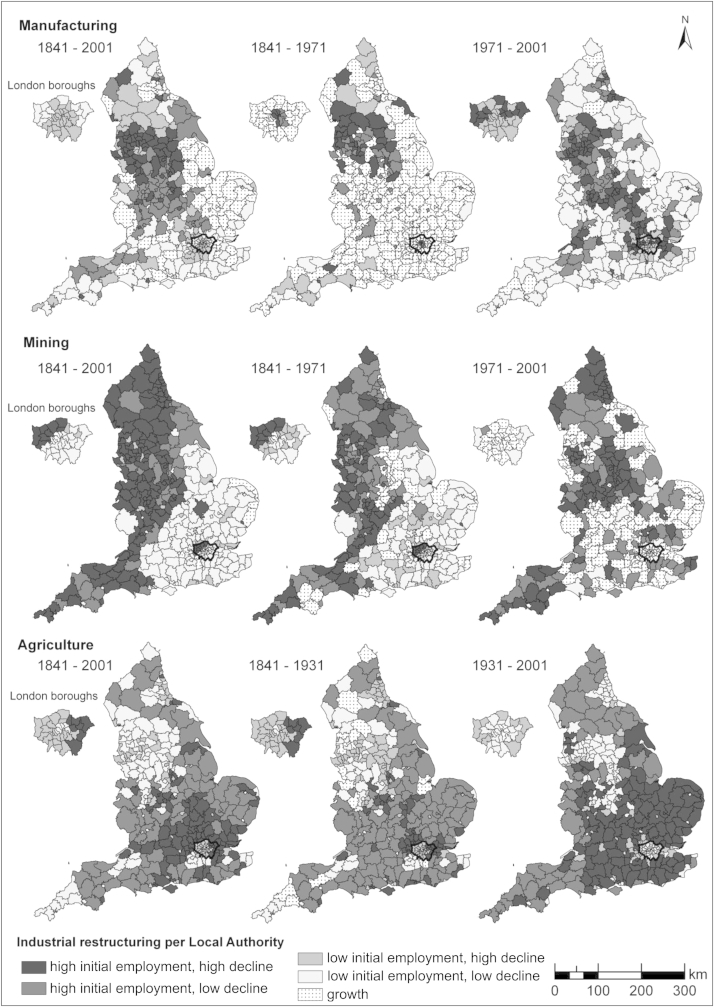
The spatial distribution of industrial restructuring across English Local Authorities, 1841–2001.

Table 2 shows changes in employment across the time periods studied. There was an overall decline for all sectors and across all time periods, except for manufacturing between 1841 and 1971, which reflects the growth in prosperity between the 1950s and early 1970s. Fig. 1 maps the derived measures of industrial decline. Across all time periods, areas with high initial employment in manufacturing and high declines in this sector are particularly concentrated in the North West and partly in the Midlands regions. High initial employment and high decline in mining was particularly prominent in the North, and partly in the Midlands and the South West. Agricultural employment particularly declined in more southerly districts.

Table 3 summarises the direction of effect of unadjusted associations between individual levels of PA and employment change per district. Most associations indicated low levels of PA to be associated with high employment decline, with the majority of statistically significant results being for domestic and recreational PA. Contrary to expectations, there was little evidence of the strength or direction of relationships differing by gender.

In regression modelling, all of the individual covariates showed associations with the PA outcomes, with PA being lower in amongst older participants, women (except for domestic PA), non-whites, those reporting limiting long term illness, and those assessing their health as either ‘bad or very bad’ or ‘poor’ compared to good (results not presented). After adjustment for these covariates, the associations with industrial decline from the regression models are presented in Table 4.

Although many of the associations did not reach statistical significance, Table 4 shows that in comparison to areas of growth, there were generally higher odds for low total PA in LADs with high initial employment, followed by employment declines in manufacturing and mining. In areas characterised by declines in manufacturing employment, residents were generally more likely to report low levels of occupational PA for all time periods and irrespective of whether initial employment was high or low. There were few associations between occupational PA and change in mining employment, though participants living in areas with declines in agricultural employment between 1931 and 2001 were actually less likely to report low levels of occupational PA. The odds of reporting low domestic activity were generally increased for participants in areas with employment decline in the manufacturing sector between 1841 and 2001, and across all three sectors between 1841 and 1971. Participants living in areas with declines in agricultural employment between 1931 and 2001 were however significantly less likely to report low levels of domestic activity.

For the recreational PA and walking domains, declines coupled with high initial employment in manufacturing were associated with higher odds of reporting low PA. Again for both domains, respondents living in areas where employment in mining had declined were more likely to report low PA, with this association particularly strong for recreational activity and employment change between 1841 and 2001. In comparison to areas of growth, residents of districts characterised by agricultural employment decline showed predominantly reduced odds for reporting low recreational activity, but increased odds for reporting low walking activity. Many of these associations were, however, not statistically significant.

After additional adjustment for individual income (on-line supplementary Table 5), associations with the area-based measures of industrial decline were slightly attenuated, although the results did not substantially differ from those presented in Table 4.

## Discussion

To our knowledge this is the first study linking different activity domains from a large national health survey to the historical context of deindustrialisation. Although, partly as a result of the number of tests we undertook, our findings were somewhat equivocal, they provide some evidence that a history of industrial decline in an area may be associated with low levels of PA. In particular, residents of districts characterised by high employment in the manufacturing sector during the period of strong industrialisation in the mid-19th century were more likely to report low PA across all five activity domains and over the studied time periods. Declining mining employment was generally associated with a decreased likelihood of high total, recreational and walking activity. Residents in areas of agricultural decline were particularly prone to reporting low walking activity.

Our findings show no evidence of a compensatory effect between the different types of PA and we found the nature of the relationships to be generally similar for men and women. This is interesting insofar as the decline in occupational PA associated with heavy manual work primarily concerns men. Perhaps living in an area with an industrial heritage may promote non-active behaviours for the whole of the population, and factors related to the socio-cultural history of areas may shape beliefs and attitudes towards PA. Certainly social and cultural backgrounds of individuals and families are known to establish PA related norms and role models which affect social environments of communities as well as those entire areas (Berger & Peerson, 2009; Fischbacher, Hunt, & Alexander, 2004). Furthermore, widening socio-economic inequalities and unequal opportunities may promote the development of environments, which increasingly hamper sufficiently active lifestyles. Although declining levels of PA and their adverse effects on health are found across the whole of the society, they become more common at the lower end of the socio-economic strata because protective factors such as better job opportunities and living conditions become less obtainable for both men and women (Ball et al., 2010; Wilkinson & Pickett, 2010).

After adjustment for individual income we found that associations with the area-based measures of industrial decline were slightly attenuated. This is as expected given that income was statistically significantly associated with all our area-based measures of industrial decline (*p* < 0.001). Furthermore, information on income was only available for 80.5% of our sample, and hence the statistical power of these additional models was reduced. As the results did not differ substantially from the results without adjusting for household income, we did not run additional models with imputed data or modelled missingness, particularly as this might increase bias (Vogl, Wenig, Leidl, & Pokhrel, 2012).

Due to the cross-sectional nature of our outcome variable, it is not possible to determine if the causal mechanisms underlying the associations with industrial change that we have observed may be mediated by present-day socio-economic circumstances, although the theory underlying our analysis does not require this. Nevertheless, the persistence of association with the area based measures after control for individual income suggests that area effects are at least partially independent of individual circumstances.

This study has a number of strengths and weaknesses. Strengths include the large, nationally representative and geographically heterogeneous samples of the HSE (Craig & Mindell, 2008; Craig et al., 2009), and the fact that the HSE includes information on five key domains of PA. Since our health related outcome was measured across multiple domains and related to different macroeconomic time periods we were also able to extend earlier work on the impact of industrial decline on population health (Mitchell et al., 2000). In particular the availability of data from the Great Britain Historical GIS project allowed us to analyse population census data collected over a period of a century and a half, yet standardised to a set of modern day units.

In terms of weaknesses, our outcome variables did not include information on changes in levels of PA over time and further work, using a longitudinal measure of PA, will be required to better understand possible causal pathways between declining levels of PA and the transformation of occupational structures. The information on the PA outcomes is largely based on self-report and several assumptions underlying the intensity level classification which make the outcomes susceptible to error (Adams et al., 2005). Furthermore, all PA measures collected in the HSE considered only bouts of at least 30 min' duration, which will have resulted in an underestimation of activity for respondents who undertook many shorter activities. Most transport-related walks, for example, do not amount to 30 min; yet even 10 min of activity have beneficial effects for health (Warburton et al., 2006b). Research from Canada provides evidence that walking to transit can account for 25% of the recommended volume of PA per day (Morency, Trépanier, & Demers, 2011).

One limitation of the data provided by the Great Britain Historical GIS project is that the standardisation of economic data covering a period of 160 years required the matching of different occupational classifications, which resulted in relatively simple categorisation of the employment sectors. Further, estimates of employment change in several LADs in the 1960s were most likely severely understated due to the conversion of population counts between different geographical units, and mining did not exist as a separate category in the 1981 census. Both factors meant we were not able to look at change for time points in the decade before and after 1971. Since the data we analysed did not comprise information on unemployment rates across the three sectors of interest, we did not consider issues related to the potential impact of hidden unemployment (Beatty & Fothergill, 1996). For example, it has been argued that pre-1997 governments were using incapacity benefits to exclude former miners from the unemployment total (Beatty & Fothergill, 1996; Haynes, Bentham, Lovett, & Eimermann, 1997), even though many may have been healthy enough to undertake light industrial work. It would have been interesting to explore whether the relationship between unemployment rates, incapacity benefits, and PA show comparable associations to those reported here.

Our modelling approach involved many comparisons and is therefore susceptible to the problems of multiple hypotheses testing where the probability of detecting statistically significant false positive or negative effects increases with each additional test (Shaffer, 1995). Although we present tests for trend in our models, this does not necessarily provide evidence of dose–response relationships as are categories of industrial decline do not fall into a single scale. While we present results ordered according to level of initial employment, we could alternatively order according to employment change.

In our analysis we do not attempt to link levels of PA with objective health outcomes such as cardiovascular disease risk factors. It may be that disparities in PA associated with industrial decline are greater than they are for associated health outcomes, as there is some evidence that occupational PA may not confer the same health benefits as recreational activity (e.g. Gutierrez-Fisac et al., 2002; Pols, Peeters, Twisk, Kemper, & Grobbee, 1997). If this were the case, then declines in occupational activity would not need to be compensated by corresponding increases in recreational activity for health to be maintained. Amongst a sample of French working adults Oppert et al. (2006) found that recreational activity was protective for a range of cardiovascular risk factors including weight status, body fat, blood pressure, and triglycerides, while occupational activity was associated with a higher risk for most factors. Interestingly, the elevated risk disappeared in that study after adjustment for educational attainment, suggesting that the evidence in this field might be limited by presence of residual confounding associated with unmeasured social-class related health behaviours.

We chose not to adjust for neighbourhood socioeconomic status (SES) or individual employment status, both of which have been associated with PA in previous research (e.g. Boone-Heinonen et al., 2011; Popham & Mitchell, 2007). Our decision was primarily based on the fact that both are, at least in part, a consequence of industrial decline and hence we might expect them to sit as mediators on the causal pathway between industrial decline and PA (Rind & Jones, in press). Given that our outcome measure (PA) is measured at a single time point we are unable to unpick the causal flow in our analysis. However, we did investigate associations with these measures (results not presented), and as expected neighbourhood SES and individual employment status were both highly statistically significantly associated with most of our measures of deindustrialisation (*p* < 0.001). Nevertheless, we present our results after adjustment for household income, which was strongly associated with both employment status (*p* < 0.001) and area SES (*p* < 0.001). We believe that additional adjustment for individual employment status and neighbourhood SES would result in over-adjustment bias, as described by Schisterman, Cole, and Platt (2009).

This study focuses on England and the results may not be applicable to other settings. However, the country provides a good case study as activity levels are relatively low and processes of deindustrialisation have been particularly pronounced (Bazen & Thirlwall, 1997; Sjöström et al., 2006). It is well known that other countries have experienced a similar history of declining levels of PA and industrial decline (Brownson, Boehmer, & Luke, 2005; Muntner et al., 2005; Sobek, 2006), and replication of our analysis in these other settings is required.

In line with prior research, we conclude that industrial decline may be an important factor in explaining inequalities in health and health related behaviours such as PA and that there is some evidence that processes of deindustrialisation may be contributing to observed disparities in PA. Our research cannot determine what the mechanisms leading to this observation may be. We suggest qualitative research may help to deepen our understanding of way by which local physical, social, cultural, political and economic conditions shaped by a history of industrial decline may influence PA behaviours. This work should focus on socio-cultural factors relevant to the context of deindustrialisation as this may facilitate the development of effective interventions to increase population levels of PA in areas with an industrial heritage.

## Figures and Tables

Data source: Great Historical GIS Project, 1841 – 2001.

**Table 1:** Health Survey for England 2006 and 2008 – descriptive statistics for the physical activity outcomes.

		Men		Women	
		*n* = 12,255		*n* = 15,159	
Outcome variables		*n*	%	*n*	%
Total physical activity	Less active	3767	30.7	5537	36.5
	Moderately active	3632	29.6	5102	33.7
	Highly active	4856	39.6	4520	29.8
Occupational activity	Non-active	7209	58.8	10,275	67.8
	Less active	2722	22.2	3221	21.2
	Moderately active	2324	19.0	1663	11.0
Domestic activity	Non-active	3541	28.9	4401	29.0
	Less active	1503	12.3	1074	7.1
	Moderately active	7211	58.8	9684	63.9
Recreational activity	Non-active	5841	47.7	8259	54.5
	Less active	615	5.0	833	5.5
	Moderately active	1403	11.4	2234	14.7
	Highly active	4396	35.9	3833	25.3
Walking activity	Less active	8820	72.0	11,407	75.2
	Moderately active	1682	13.7	1722	11.4
	Highly active	1753	14.3	2030	13.4

**Table 2:** Initial employment and employment change in manufacturing, mining and agriculture, England 1841–2001

Employment type	Year (baseline)	% Employment in baseline year	Period of change	% Change
Manufacturing	1841	31.6	1841–2001	−50.6
	1971	35.0	1841–1971	10.8
	2001	15.6	1971–2001	−55.4
	1841	3.1	1841–2001	−90.3
Mining	1971	1.5	1841–1971	−51.6
	2001	0.3	1971–2001	−80.0
	1841	20.7	1841–2001	−92.3
Agriculture	1931	6.8	1841–1931	−67.1
	2001	1.6	1931–2001	−76.5

**Table 3:** Direction of associations between levels of physical activity and industrial decline across English Local Authorities.

Period of employment change	Sex	Results of the Pearson Chi-square tests (*X*^2^-value, level of significance, direction of effect [γ]^a^)				
		Physical activity domain				
		Total	Occupational	Domestic	Recreational	Walking
*% Change in manufacturing employment*						
1841–2001	Male	7.8 +	22.0** −	32.6** −	21.9** +	15.0** +
	Female	1.9 −	5.5 −	30.6** −	14.9* −	2.3 +
1841–1971	Male	5.7 +	2.8 −	11.2* −	5.0 −	10.7* +
	Female	2.1 −	1.9 −	11.7* +	7.7 −	8.3 +
1971–2001	Male	7.6 −	13.4** −	79.0** −	35.8** +	5.3 −
	Female	4.5 −	14.0** −	37.5** −	26.6** −	2.3 +
*% Change in mining employment*						
1841–2001	Male	14.3** −	2.1 −	9.4 −	34.4** −	21.1** −
	Female	20.8** −	5.3 −	5.3 +	76.6** −	27.8** −
1841–1971	Male	4.8 −	12.3* −	30.2** −	10.2 +	2.5 +
	Female	1.4 −	9.1 −	18.9** −	8.0 −	5.7 −
1971–2001	Male	3.1 −	5.6 +	13.0* +	30.8** −	10.5* −
	Female	11.0* −	4.4 −	17.1** +	10.3 −	18.9** −
*% Change in agricultural employment*						
1841–2001	Male	0.5 −	0.5 −	10.6** −	14.2** +	2.3 −
	Female	2.3 −	<0.1 −	2.2 −	9.6* −	3.3 −
1841–1931	Male	12.1* −	5.9 −	61.3** −	20.5** +	9.5* −
	Female	9.3 −	3.7 −	42.0** −	50.8** −	8.1 −
1931–2001	Male	12.4* +	3.1 −	76.9** +	27.6** +	14.9** +
	Female	18.8** +	3.4 +	55.6** +	43.7** +	19.2** +

a Pearson chi-square:**p < 0.01, *p < 0.05, all other values are not statistically significant; direction of the gamma coefficient: − indicates lower levels of physical activity associated with higher employment decline; + indicates higher levels of physical activity associated with higher employment decline.

**Table 4:** Summary of odds ratios from ordinal regression predicting lower physical activity^a^ for five physical activity domains by types and periods of industrial decline (not adjusted for equalised household income).

Odds ratio (OR)^b^	Total physical activity OR (*n* = 27,414)			Occupational physical activity			Domestic physical activity			Recreational physical activity			Walking activity		
	Time period			OR (*n* = 27,414)			OR (*n* = 27,414)			OR (*n* = 27,414)			OR (*n* = 27,414)		
	1841–2001	1841–1971	1971–2001	1841–2001	1841–1971	1971–2001	1841–2001	1841–1971	1971–2001	1841–2001	1841–1971	1971–2001	1841–2001	1841–1971	1971–2001
*Manufacturing*^c^															
High initial employment, high decline	1.12*	0.96	1.13	1.16*	1.09	1.25	1.09	1.03	1.07	1.13	1.08	1.04	1.07	0.89	1.07
High initial employment, low decline	1.08	1.09*	1.07	1.13	1.09	1.03	1.01	1.02	0.90	1.00	1.14*	1.08	1.05	1.04	1.09
Low initial employment, high decline	0.98	0.81*	0.99	1.35**	0.91	1.37	1.25**	0.91	1.19	0.87*	0.87	0.84	0.76**	0.61**	0.83
Low initial employment, low decline	0.99	0.84*	0.95	1.15*	0.87	1.04	1.22**	0.82	0.95	0.90	0.97	0.91	0.88*	0.87	0.93
Direction and *p*-value for trend	+**	ns	+**	ns	ns	+**	ns	ns	ns	+**	+*	+**	+*	ns	+**
*Mining*^c^															
High initial employment, high decline	1.13	1.07	1.03	0.87	0.99	0.92	0.87	1.09	0.87**	1.49**	1.10	1.17**	1.19	1.03	1.17**
High initial employment, low decline	1.04	1.07	1.00	0.84	0.91	1.02	0.79*	1.05	0.94	1.32*	1.17*	0.91	1.09	1.10	1.04
Low initial employment, high decline	1.10	1.03	nd	0.94	1.04	nd	0.89	1.17*	nd	1.53**	1.05	nd	1.13	0.94	nd
low initial employment, low decline	1.04	1.00	1.04	0.88	0.94	0.94	0.96	1.18**	1.05	1.25*	0.95	1.07	1.04	0.98	1.09
Direction and *p*-value for trend	+**	ns	ns	ns	ns	ns	−*	ns	−**	+**	+**	+*	+**	ns	+**
*Agriculture*^c^															
High initial employment, high decline	1.02	1.07	0.95	0.95	0.95	0.70*	1.00	1.14	0.63**	0.93	0.99	0.77	1.11	1.12	1.40*
High initial employment, low decline	0.93	1.06	0.83	0.87*	0.92	0.55**	0.90	1.07	0.52**	0.97	0.92	0.77	1.09	1.07	1.30
Low initial employment, high decline	1.05	1.13	0.97	1.05	1.05	0.73*	1.07	1.17	0.68*	1.03	1.08	0.76	1.06	1.06	1.30
Low initial employment,low decline	Baseline	1.03	1.03	Baseline	0.97	0.73*	Baseline	1.12	0.67**	Baseline	0.92	0.93	Baseline	0.95	1.44*
Direction and *p*-value for trend	ns	ns	−**	ns	ns	+*	ns	ns	−*	ns	ns	−**	ns	+*	ns

a Coding for the outcome variables: total physical activity and walking activity: 1 = less active, 2 = moderately active, 3 = highly active; occupational and domestic activity: 1 = non-active, 2 = less active, 3 = moderately active; recreational activity (sport): 1 = non-active, 2 = less active, 3 = moderately active, 4 = highly active.

b **p < 0.01,*p < 0.05, all other values are non-significant. OR > 1.00 indicates increased likelihood of lower physical activity; OR < 1.00 indicates decreased likelihood of lower physical activity; positive trend: lower physical activity associated with higher initial employment and higher employment decline; negative trend: higher physical activity associated with higher initial employment and higher employment decline.

c Baseline: employment growth; exception: models “Agriculture 1841–2001”: low initial employment, low decline as baseline since there were no areas of growth; nd = no districts observed in cell.
