# Supplementary material for: How is post-industrial decline associated with the geography of physical activity? Evidence from the Health Survey for England
Source: Soc Sci Med. 2014 Mar;104(100):88–97. doi: 10.1016/j.socscimed.2013.12.004 (PMC3988884; doi:10.1016/j.socscimed.2013.12.004)
Supplement: Supplementary file 1 [file mmc1.docx]

Supplementary material

Table 5

Summary of odds ratios from ordinal regression predicting lower physical activity^†^ for five physical activity domains by types and periods of industrial restructuring, adjusted for equalised household income

^†^Coding for the outcome variables:

Total physical activity and walking activity: 1 = less active, 2 = moderately active, 3 = highly active; occupational and domestic activity: 1 = non-active, 2 = less active, 3 = moderately active; recreational activity (sport): 1 = non-active, 2 = less active, 3 = moderately active, 4 = highly active.

^‡^***p* < 0.01,**p* < 0.05, all other values are non-significant.

OR > 1.00 indicates increased likelihood of lower physical activity; OR < 1.00 indicates decreased likelihood of lower physical activity; positive trend: lower physical activity associated with higher initial employment and higher employment decline; negative trend: higher physical activity associated with higher initial employment and higher employment decline.

^¥^Baseline: employment growth; exception: models “Agriculture 1841 – 2001”: low initial employment, low decline as basline since there were no areas of growth; nd = no districts observed in cell.
